# Nicotine exacerbates diabetic nephropathy through upregulation of Grem1 expression

**DOI:** 10.1186/s10020-023-00692-9

**Published:** 2023-07-06

**Authors:** Jianning Chen, Haiting Xiao, Rui Xue, Vinod Kumar, Rukhsana Aslam, Syed Faizan Mehdi, Huairong Luo, Ashwani Malhotra, Xiqian Lan, Pravin Singhal

**Affiliations:** 1 Key Laboratory of Luzhou City for Aging Medicine, Department of Pharmacology, School of Pharmacy, Southwest Medical University, Luzhou, 646000 Sichuan China; 2 Affiliated Mental Health Center and Hangzhou Seventh People’s Hospital, Zhejiang University School of Medicine, Hangzhou, 310000 Zhejiang China; 3 Feinstein Institute for Medical Research and Donald and Barbara Zucker School of Medicine at Hofstra/Northwell, Manhasset, NY 11030 USA

**Keywords:** Grem1, Nicotine, Diabetic nephropathy, TGF-β, Therapeutic target

## Abstract

**Background:**

Diabetic nephropathy (DN) is a major complication of diabetes mellitus. Clinical reports indicate that smoking is a significant risk factor for chronic kidney disease, and the tobacco epidemic exacerbates kidney damage in patients with DN. However, the underlying molecular mechanisms remain unclear.

**Method:**

In the present study, we used a diabetic mouse model to investigate the molecular mechanisms for nicotine-exacerbated DN. Twelve-week-old female mice were injected with streptozotocin (STZ) to establish a hyperglycemic diabetic model. After four months, the control and hyperglycemic diabetic mice were further divided into four groups (control, nicotine, diabetic mellitus, nicotine + diabetic mellitus) by intraperitoneal injection of nicotine or PBS. After two months, urine and blood were collected for kidney injury assay, and renal tissues were harvested for further molecular assays using RNA-seq analysis, real-time PCR, Western blot, and immunohistochemistry. In vitro studies, we used siRNA to suppress Grem1 expression in human podocytes. Then we treated them with nicotine and high glucose to compare podocyte injury.

**Result:**

Nicotine administration alone did not cause apparent kidney injury, but it significantly increased hyperglycemia-induced albuminuria, BUN, plasma creatinine, and the kidney tissue mRNA expression of KIM-1 and NGAL. Results from RNA-seq analysis, real-time PCR, Western blot, and immunohistochemistry analysis revealed that, compared to hyperglycemia or nicotine alone, the combination of nicotine treatment and hyperglycemia significantly increased the expression of Grem1 and worsened DN. In vitro experiments, suppression of Grem1 expression attenuated nicotine-exacerbated podocyte injury.

**Conclusion:**

Grem1 plays a vital role in nicotine-exacerbated DN. Grem1 may be a potential therapeutic target for chronic smokers with DN.

**Supplementary Information:**

The online version contains supplementary material available at 10.1186/s10020-023-00692-9.

## Introduction

Diabetes mellitus is a global epidemic that is increasing yearly, seriously threatening human health. An estimated 463 million people worldwide had type 2 diabetes in 2019, according to the International Diabetes Federation. This number is expected to rise to 700 million by 2045 (Saeedi et al. 2019). A nationally representative survey of over 75,000 adults published in 2020 showed that the prevalence of diabetes in China was 11.2% (Li et al. 2020). Diabetic nephropathy (DN) is a common microvascular complication of diabetes (20–40%) (Warren et al. 2019). It manifests in progressive proteinuria, decreased glomerular filtration rate (GFR), and glomerular hypertension with higher cardiovascular morbidity and mortality (Zhang et al. 2016; Ma et al. 2019). DN's etiology and molecular mechanisms are unclear, making it difficult to prevent and treat. An increasing prevalence of diabetes and associated high morbidity and mortality have led to the current management of diabetic kidney patients poses an enormous socioeconomic burden on society (Saeedi et al. 2019). Henceforth, there is an urgent need to improve the mechanistic understanding of DN and to develop new and practical therapeutic approaches to prevent or delay the progression of DN.

The tobacco epidemic is a significant public problem in most developing countries and is associated with high morbidity and mortality. The tobacco epidemic is also a significant public health problem. For example, China is the world's largest tobacco producer and consumer (The Writing Committee of 2020 Report on Health Hazards of Smoking in China 2021) and inhabits more than 300 million smokers; there are more than 1 million deaths yearly result from smoking-related diseases, including more than 100,000 deaths from secondhand smoke exposure (Wang et al. 2018).

Smoking has been reported to be an important risk factor for diabetes, and smokers have twice the risk of developing diabetes as nonsmokers (Mardarowicz et al. 2010). Animal experiments have also confirmed that cigarette smoking aggravates diabetic kidney injury in rats and mice (Jiang et al. 2019; Jaimes et al. 2021). Recent studies demonstrate that nicotine, one of the highly active compounds in cigarette smoke, contributes to the pathogenesis of smoking-mediated kidney dysfunction (Hukkanen et al. 2005; Rezonzew et al. 2012). Nicotine mediates its effects via activating muscle and neuronal nicotinic acetylcholine receptors (nAChRs) (Albuquerque et al. 2009). Studies from our group and others have confirmed that the nAChRs are expressed in non-neuronal cells, including kidney cells (podocytes, tubular epithelial cells, and mesangial cells), endothelial cells, and vascular smooth muscle cells. We found that nicotine promoted podocyte apoptosis by activating MAPK kinases and enhancing oxidative stress (Lan et al. 2016). It is reported that short-term stimulation of tubular epithelial cells with a high dose of nicotine (200–400 μM) causes apoptosis or epithelial-mesenchymal transition (EMT) through enhanced ROS generation (Arany et al. 2012; Kim et al. 2016), while long-term stimulation with a low dose of nicotine (1–10 μM) increases proliferation through activation of AKT (Chang and Singh 2018). For mesangial cells, nicotine stimulation activates the TGF-β or Wnt/β-catenin pathway, which promotes the cell proliferation and production of extracellular matrix proteins (Jaimes et al. 2007, 2009; Hua et al. 2010; Lan et al. 2018). These studies show that nicotine stimulation causes injuries to kidney cells, leading to renal dysfunction. However, it is still unclear whether nicotine plays a vital role in promoting DN, and the molecular mechanism of nicotine in promoting DN is still unclear.

As a high-throughput sequencing technology developed rapidly in recent years, RNA sequencing (RNA-seq) technology has been widely used to study gene functions. It has been a powerful method for studying the molecular mechanism of specific biological processes and diseases (Lv et al. 2020). Using RNA-seq, many critical genes related to the pathogenesis of kidney disease have been identified (Yu et al. 2020; Hassanzadeh and Wang 2021; Kilari et al. 2021; Tang et al. 2021; Zheng et al. 2021).

In this study, we performed RNA-seq to analyze the gene expression changes in mouse kidneys caused by nicotine-exacerbated DN. We identified Grem1 as a vital gene promoting kidney damage in response to nicotine in the diabetic milieu. We also examined the expression and role of Grem1 and its downstream genes belonging to the TGF-β pathway in nicotine-exacerbated DN.

## Materials and methods

### Animals and treatments

All animal experiments complied with Institutional Animal Care and Use Committee (IACUC)-approved protocols. The mice were housed within the rodent holding facilities at the Feinstein Institute for Medical Research (Northwell Health) in Manhasset, New York. It is under temperature, light, and humidity control. Adequate food, water, and bedding were provided. Unless otherwise mentioned, all animal procedures and treatments were performed as described in our previous publications (Wen et al. 2019; Lan et al. 2020). To generate Streptozotocin (STZ)-induced diabetic mouse model, 40 female mice (12-week-old, 20–25 g) on FVB/N background were fasted but given water freely for 18 h and then were injected intraperitoneally with streptozotocin (Sigma-Aldrich, St. Louis, MO, USA), freshly dissolved in sterile PBS at a dose of 100 mg/kg body weight for three consecutive days. As untreated control, another 14 mice received sterile PBS only in the same manner. Four days after the last injection, the blood glucose levels of the mice were measured. Mice with glucose levels above 200 mg/dL were considered successful hyperglycemic (diabetic model, DM) and used for further experiments. After four months, the control group (14 mice survived) and diabetic mice (21 survived mice and showed hyperglycemia) were further divided into subgroups, as follows: (1) control (Con) group; (2) nicotine (Nic) group; (3) diabetic mellitus (DM) group; (4) Nic + DM (N + D) group. Mice in the Nic (9 mice) and Nic + DM (13 mice) groups were administered an intraperitoneal injection of nicotine (1 mg/kg every other day). In contrast, mice in the Con (5 mice) and DM (9 mice) groups were parallelly administrated with PBS. After two months, all 5 mice in the Con group survived; 7 mice in the Nic group, 6 in the DM group, and 8 in the Nic + DM group survived. The urine and blood samples were collected at the end of the experimental period, and the mice were sacrificed with CO_2_. Blood was collected, and kidney samples were harvested. The mouse administration procedure is summarized in Additional file 1: Fig. S1.

Albuminuria was assessed using a mouse albumin ELISA kit (Bethyl Lab, Hamburg, Germany). Blood urea nitrogen (BUN) was determined using the Urea Nitrogen (BUN) Colorimetric Detection Kit (Thermo Fisher Scientific, Waltham, MA). Creatinine was measured using the Creatinine Assay Kit (ab204537, Abcam, Waltham, MA).

### Serum cotinine measurement

Eight mice (12-week-old) were administered an intraperitoneal injection of nicotine (1 mg/kg). As a parallel control, another 6 mice were injected with PBS. After 30 min, blood samples were collected from the submandibular (facial) vein. Immediately after collection, blood samples were spun (3000 *g* for 10 min), and then the plasmas were collected and frozen at − 80 °C until analysis. Cotinine levels of the plasmas were measured with an ELISA kit (Cat# EA100901, OriGene, Rockville, MD), following the manufacturer's instructions.

### Culture of human podocytes

Human podocytes were cultured as previously reported (Lan et al. 2016; Wen et al. 2019). Briefly, immortalized human podocytes were cultured in a growth medium containing RPMI 1640 supplemented with 10% fetal bovine serum,1 X penicillin–streptomycin, one mM L-glutamine, and 1X insulin, transferrin, and selenium (ITS) (Invitrogen, Grand Island, NY) at the permissive temperature (33 ℃). When cells reached about 80% confluence, they were transferred to 37 ℃ for seven days of differentiation in an ITS-free medium.

### Apoptosis determination

TUNEL staining. In mouse tissues, apoptotic cells were assayed using TUNEL staining with Click-iT™ TUNEL Colorimetric IHC Detection Kit (Thermo Fisher Scientific, C10625). In in vitro studies, we determined cellular apoptosis using Hoechst33342 staining, as described in previous publications (Lan et al. 2016; Wen et al. 2019). Cells with condensed and fragmented nuclei were identified as apoptotic cells. The nuclei of healthy cells appeared blue (TUNEL-negative), and the nuclei of apoptotic cells were stained brown (TUNEL-positive) after TUNEL staining.

### Real-time PCR

Real-time PCR was performed as previous descriptions (Lan et al. 2013, 2016; Wen et al. 2019). Total RNA was isolated from mouse kidney samples using TRIzol reagent (Invitrogen). Five micrograms of total RNA were reverse-transcribed using the first-strand synthesis system (Invitrogen). Real-time PCR was performed in a Prism 7900HT sequence-detection system (Applied Biosystems, Foster City, CA, USA). Using the comparative CT method, a GAPDH internal control determined and standardized relative mRNA levels. GAPDH was used as an internal control. The primer sequences are listed in Table 1.

**Table 1:** The primers for qPCR

Gene	Forward primer	Reverse primer
Grem1	GCACATCCGAAAGGAGGAAG	ATGGATATGCAACGGCACTG
Kim-1	CCAATGGACATCGTGTCACC	GGGTCTTCTTGGAGGACGTG
NGAL	ATTTGTTCCAAGCTCCAGGGC	CCTTCAGTTCAGGGGACAGCT
Id1	CATGAACGGCTGCTACTCACG	GTCCCGACTTCAGACTCCGAG
Id4	CTGTGCCTGCAGTGCGATATG	AAAGCAGGGTGAGTCTCCAGC
Snail1	ATTCTCCTGCTCCCACTGC	GACTCTTGGTGCTTGTGGAG
GAPDH	CCATGGAGAAGGCTGGGC	CAAAGTTGTCATGGATGA

### Western blotting analysis

Western blotting was performed as described in our previous publications (Lan et al. 2013, 2016; Wen et al. 2019). The acquired images were analyzed using the NIH image program in the public domain. Primary antibodies used were rabbit anti-Grem1 (Novus Biologicals, NBP1-31150, 1:1000), rabbit anti-P-Smad2/3 (Abcam, ab63399, 1:1000), rabbit anti-P-Smad1/5/8 (Millipore Sigma, AB3848-I, 1:1000), and mouse anti-actin (Santa Cruz Biotechnology, sc-8432, 1:3000). For protein expression quantification, films were scanned using a Canon Scan 9950F scanner.

### RNA sequencing and data analysis

The extraction of total RNA has been described above. In the subsequent procedure, mRNA molecules were purified from total RNA using Oligo(dT)-attached magnetic beads. The Agilent Bioanalyzer 2100 system analyzed the cDNA libraries' qualities. RNA-seq was conducted using the BGISEQ500 platform. Clean reads were obtained from the raw data after removing the adapter, ploy-N, and low-quality reads. HISAT was used to align clean reads to the reference genome. Bowtie2 was used to align clean reads to reference gene sequence, and RSEM was used to calculate gene and transcript expression levels. The method of fragments per kilobase of transcript sequence per million base pairs (FRKM) was used to calculate the expression level of each transcript. The software DESeq2 was used to analyze the differential expression genes. The standard p-value < 0.05 and log_2_(fold change) > 0 (upregulated) or log_2_(fold change) < 0 (down-regulated) were taken to identify differentially expressed genes (DEGs). Fuzzy C-means clustering was used to identify mice's gene group responding to nicotine-exacerbated DN.

### Immunohistochemistry and histology studies

Immunohistochemical and immunofluorescent studies were performed as described previously (Jaimes et al. 2007; Lan et al. 2018). Primary antibodies were rabbit anti-Grem1 (Novus Biologicals, NBP1-31150, 1:100), rabbit anti-Nephrin (Abcam, Cambridge, MA, ab58968, 1:100), rabbit anti-P-Smad2/3 (Abcam, ab272332, 1:100), rabbit anti-P-Smad1/5/8 (Sigma-Aldrich, Burlington, MA, AB3848-I, 1:100). Periodic acid-Schiff (PAS) staining was conducted by specialists at the Pathology Department, Long Island-Jewish Hospital (Queens, NY), following standard staining protocols. Masson trichrome staining was performed by specialists at the Pathology Department, Shanghai Gefan Biotechnology Co., Ltd (Shanghai, China), following standard staining protocols.

### Statistical analysis

All data were statistically evaluated by analysis of variance (ANOVA) followed by Newman-Keuls multiple comparison tests using software (Prism 4.0, GraphPad Software). Statistical significance was considered when p values < 0.05.

## Results

### Nicotine exacerbates DN in mice

**Fig. 1:**
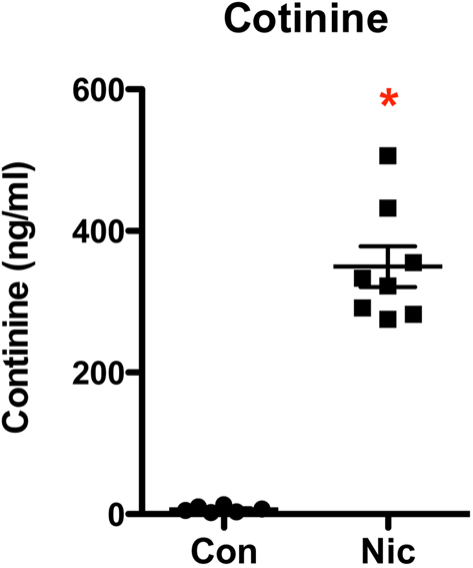
Cotinine levels in mouse serum after nicotine administration. Twelve-week-old mice were administrated with PBS or nicotine (1 mg/kg) through IP injection. After 30 min, serum samples were collected, and the cotinine levels were determined with an ELISA kit. The results (mean ± SD) from the samples of individual mice (n = 6 in the Con group, 8 in the Nic group) are displayed. *(p < 0.05) compared with the control mice

Previous studies have indicated that mouse plasma cotinine levels peak 15 to 30 min after injection (Nguyen et al. 2020; Siu and Tyndale 2007). Based on these findings, we administered mice with either PBS or nicotine (1 mg/kg) via intraperitoneal (IP) injection. We collected their plasma samples after a 30-min interval. The cotinine levels in the plasma samples were subsequently quantified using an ELISA kit. As anticipated, cotinine levels in the plasma samples from mice administered with PBS were nearly undetectable. At the same time, nicotine administration significantly increased the mean cotinine level (349.5 ng/ml ± 81.0 ng/ml, Fig. 1).

**Fig. 2:**
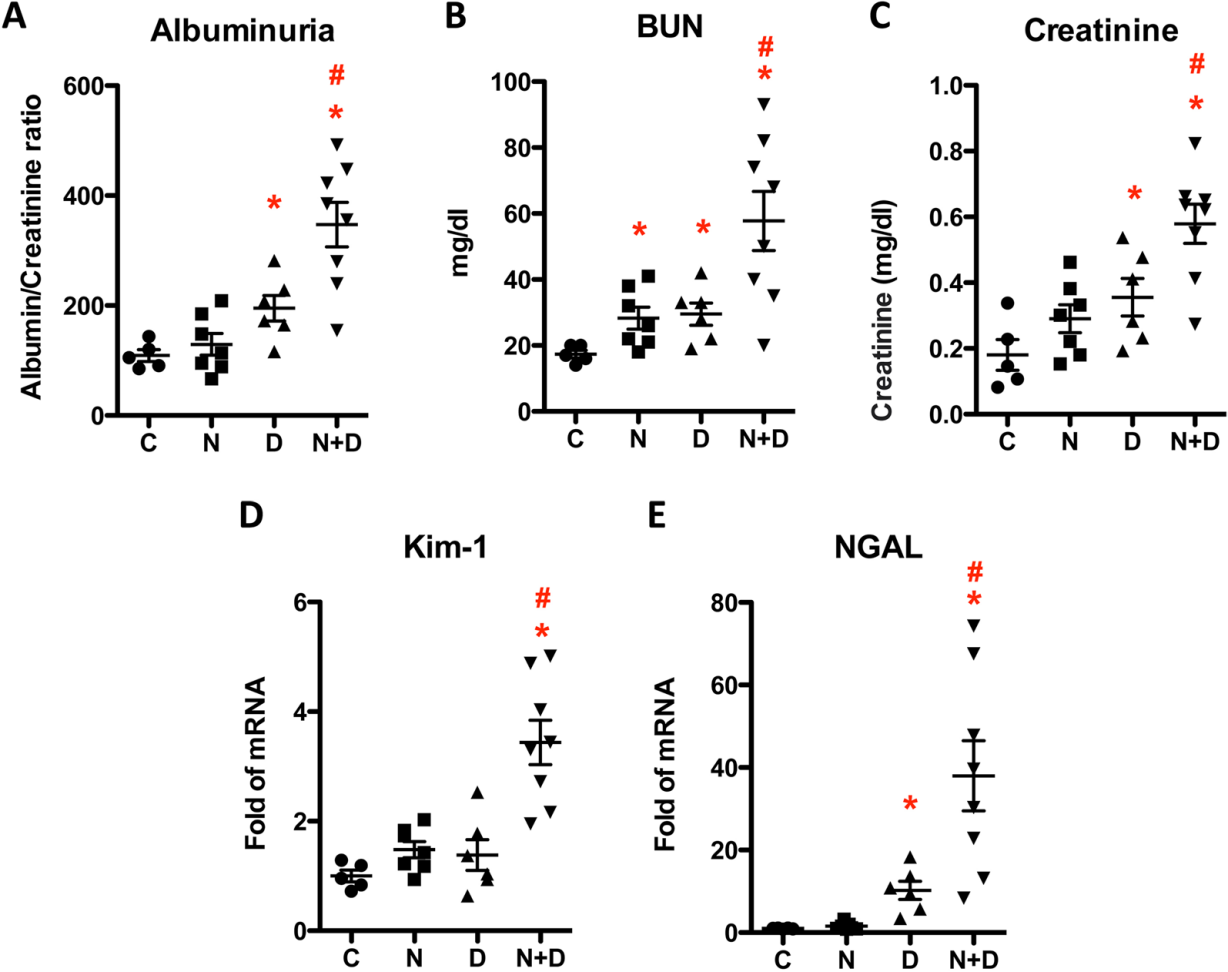
Nicotine exacerbates mouse diabetic nephropathy. Control and experimental mice were divided into four groups: (1) control (abbreviated as C) group; (2) nicotine (N) group; (3) diabetic mellitus (D) group; (4) combination of nicotine and diabetic mellitus (N + D) group. At the end of the experimental period, urine samples were collected to assay the albumin: creatinine ratio (**A**), and blood samples were collected to determine BUN (**B**) and creatinine (**C**). RNA samples were collected from the kidneys, and real-time PCR was performed to determine the mRNA expression of KIM-1 and NGAL, respectively (**D**, **E**). The results (mean ± SD) from the samples of individual mice (n = 5 in the C group, 7 in the N group, 6 in the D group, and 8 in the N + D group) were displayed. For all groups, * (p < 0.05) compared with the control mice (C) group, and # (p < 0.05) compared with the diabetic mellitus (D) group

To examine the effects of nicotine and hyperglycemia on kidney injuries, we determined albuminuria, BUN, and plasma creatinine in treated mice. The results showed that nicotine alone increased BUN but not albuminuria or plasma creatinine, whereas hyperglycemia significantly increased all (Fig. 2A–C). Interestingly, the combination of nicotine and hyperglycemia further increased albuminuria, BUN, and creatinine compared with hyperglycemia alone (Fig. 2A–C). We then detected the expression of kidney injury molecule-1 (KIM-1) and neutrophil gelatinase-associated lipocalin (NGAL), two biomarkers for kidney injury, by real-time PCR. The results showed that nicotine alone did not increase the expression of either gene. In contrast, its combination with hyperglycemia significantly increased the expression of both genes compared to hyperglycemia alone (Fig. 2D, E).

**Fig. 3:**
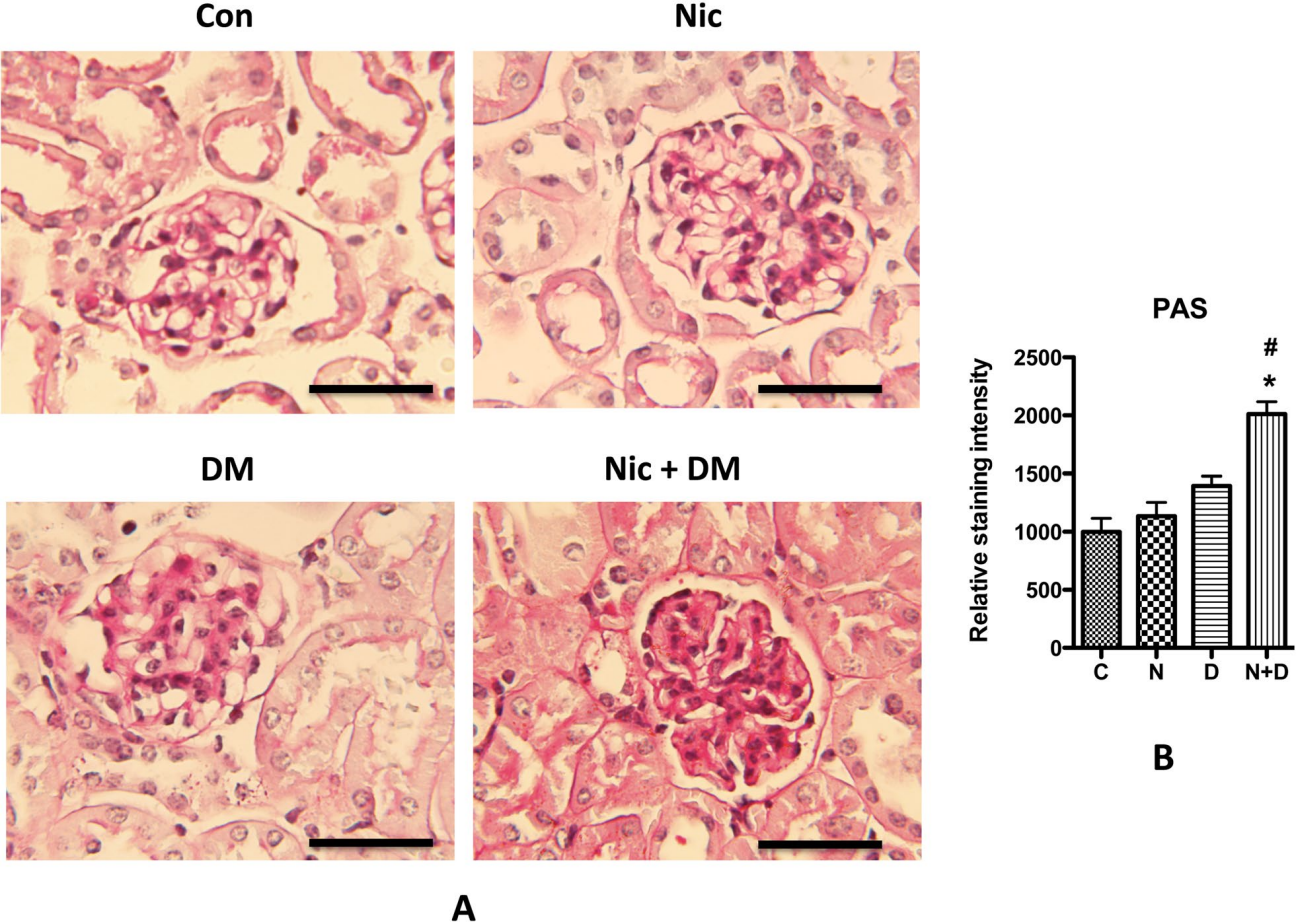
PAS staining for the kidneys of diabetic mice. **A** Periodic Acid Schiff (PAS) staining in kidneys of Con and Nic, DM, Nic + DM mice were analyzed. The scale bar is 20 μm. **B** Results (mean ± SD) represent 10 randomly and blindly selected regions, and representative microphotographs are displayed. Note: C, control; N, nicotine; D, diabetes mellitus; N + D, nicotine + diabetes mellitus. * (p < 0.05) was considered statistically significant when compared with the Con group, and # (p < 0.05) when compared with the D group

Glycogen accumulation is one of the key morphological changes in diabetic kidneys. To compare the kidney glycogen accumulation in control and experimental mice in response to nicotine and hyperglycemia, we performed PAS staining, an easy and commonly used method to identify glycogen in the diabetic kidney (Kang et al. 2005; Glastras et al. 2016). Results showed that nicotine or hyperglycemia alone did not increase the PAS staining in kidney tissues; in contrast, the combination of nicotine and hyperglycemia significantly increased the PAS staining in both glomerular and interstitial spaces, suggesting nicotine and hyperglycemia synergistically increased kidney glycogen content (Fig. 3).

**Fig. 4:**
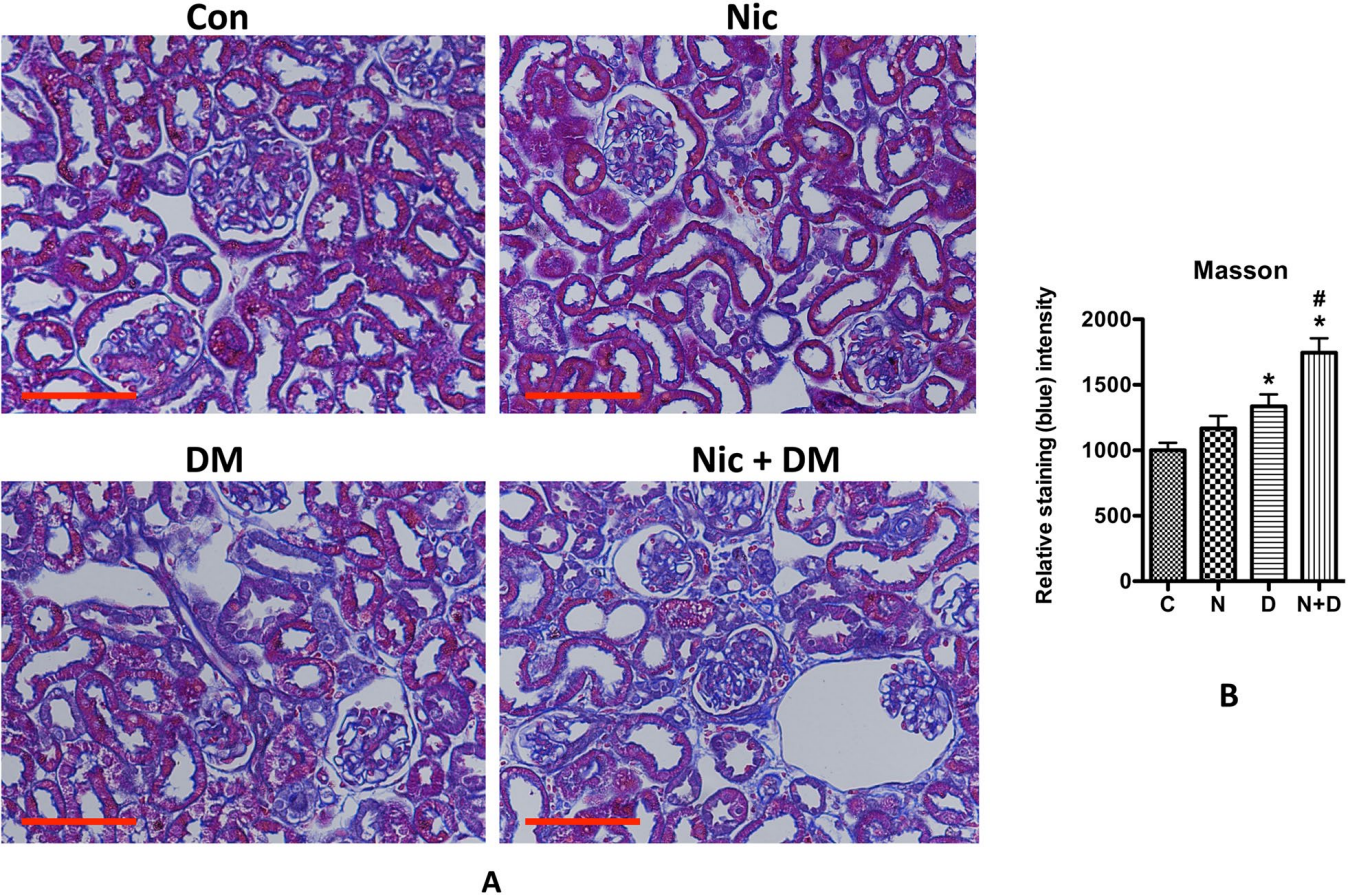
Masson trichrome staining for the kidneys of diabetic mice. **A** Masson trichrome staining in the kidneys of Con and Nic, DM, and Nic + DM mice were analyzed. The scale bar is 50 μm. **B** Results (mean ± SD) represent 10 randomly and blindly selected regions, and representative microphotographs are displayed. Note: C, control; N, nicotine; D, diabetes mellitus; N + D, nicotine + diabetes mellitus. * (p < 0.05) was considered statistically significant when compared with the Con group, and # (p < 0.05) when compared with the D group

Another significant morphological change observed in the diabetic kidney is collagen accumulation. To identify collagen in the diabetic kidney, we employed Masson trichrome staining, a commonly utilized and straightforward method (Mise et al. 2017; El-Bahy et al. 2018). The results demonstrated that nicotine alone did not increase collagen staining (blue in the Masson trichrome staining) in kidney tissues. However, STZ-induced hyperglycemia significantly enhanced collagen staining. Remarkably, the combination of nicotine and hyperglycemia further intensified collagen staining in both glomerular and interstitial region, indicating a synergistic effect of nicotine and hyperglycemia on kidney collagen content (Fig. 4).

**Fig. 5:**
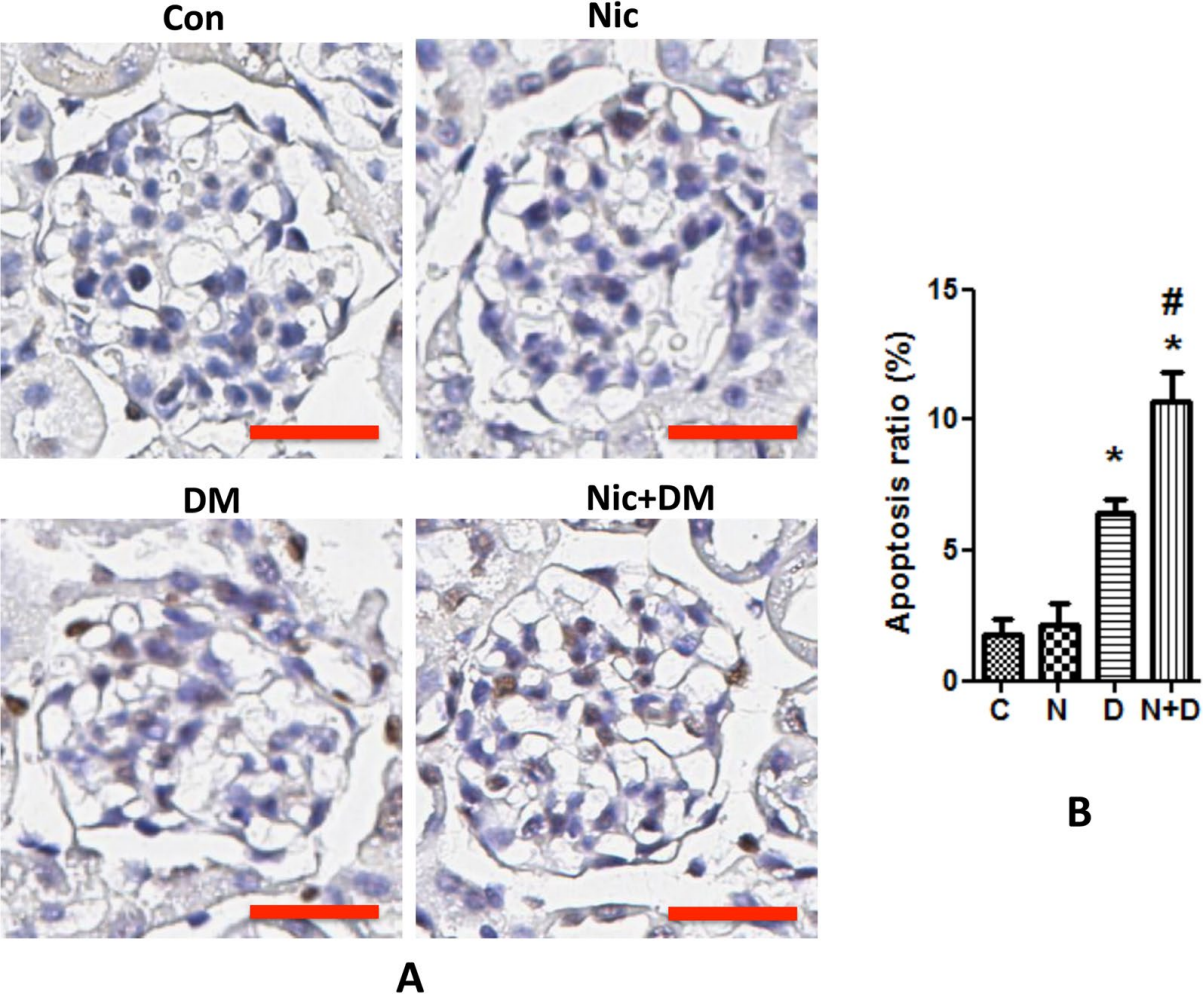
Nicotine increases apoptosis in the kidney of diabetic mice. FVB/N mice were administered nicotine or hyperglycemic (for 6 months). Then the kidney samples were collected for TUNEL staining to examine the apoptotic cells. **A** TUNEL staining in the kidneys of Con and Nic, DM, and Nic + DM mice were analyzed. Representative images are shown. The scale bar is 20 μm. **B** Results (mean ± SD) represent 10 randomly and blindly selected regions, and representative figures are displayed. * (p < 0.05) compared with the C group, and # (p < 0.05) compared with the D group. C, control; N, nicotine; D, diabetes mellitus; N + D, nicotine + diabetes mellitus

We also examined apoptosis caused by nicotine and hyperglycemia by using TUNEL staining. We found that a combination of nicotine and hyperglycemia promoted apoptosis in a more significant number of kidney cells than in either treatment alone (Fig. 5).

**Fig. 6:**
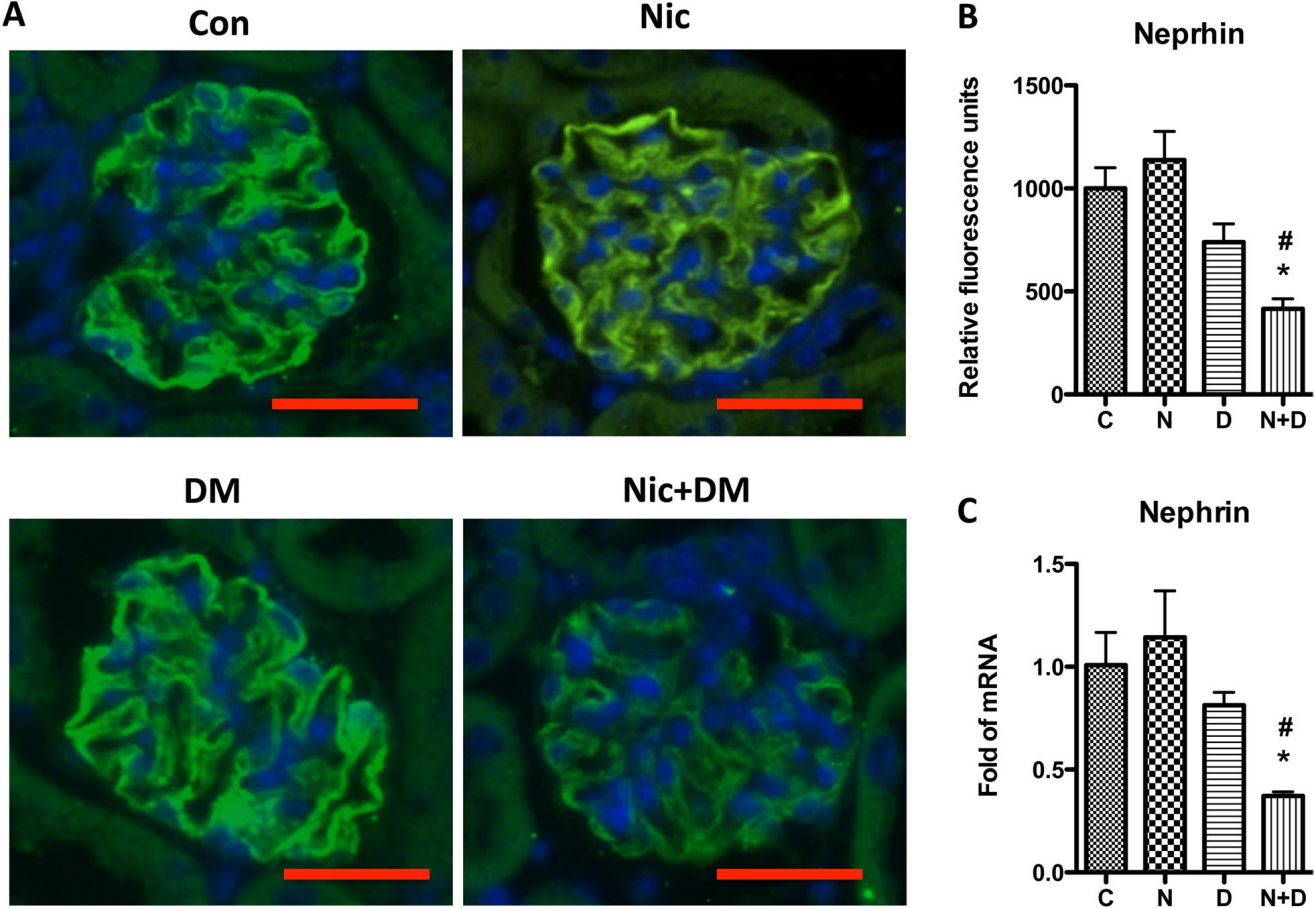
Nicotine decreases Nephrin expression in the kidney of diabetic mice. Immunofluorescence staining and q-PCR were performed to detect the Nephrin expression. **A** Immunofluorescence staining was performed to detect Nephrin protein expression in the kidneys of Con and Nic, DM, and Nic + DM mice. Representative images are shown. The scale bar is 20 μm. **B** Results (mean ± SD) represent 10 regions randomly and blindly selected from Immunofluorescence studies. **C** qPCR was performed to determine Nephrin mRNA expression in the mouse kidneys. Results (mean ± SD) from three independent samples are displayed. For all groups, * (p < 0.05) when compared with the Con group, and # (p < 0.05) compared with the DM group. Note: C, control; N, nicotine; D, diabetes mellitus; N + D, nicotine + diabetes mellitus

Nephrin is a vital slit diaphragm (SD) molecule, a principal constituent of the glomerular filtration barrier. A decreased nephrin expression is associated with podocyte injury and kidney dysfunction (Jourdan et al. 2018; Poulaki et al. 2020). We performed real-time PCR and immunofluorescence staining to examine the effects of nicotine and hyperglycemia on nephrin expression. Both modalities showed that the combined treatment (nicotine and hyperglycemia) decreased nephrin protein and mRNA expressions compared to the treatment with nicotine or hyperglycemia alone (Fig. 6).

Although typical diabetic glomerular injury (i.e., mesangial cell expansion) was not seen in any group, which might be because of the relatively shorter duration of the treatment. However, compared with the mice in nicotine or diabetic mellites groups, those in the Nic + DM group displayed some other characteristics for kidney injuries, such as proteinuria, increased BUN and blood creatinine, cell apoptosis, glycogen accumulation, and nephrin decrease. Taken together, these results demonstrated that nicotine exacerbated DN in mice.

### Transcriptome changes induced by nicotine-exacerbated DN

**Fig. 7:**
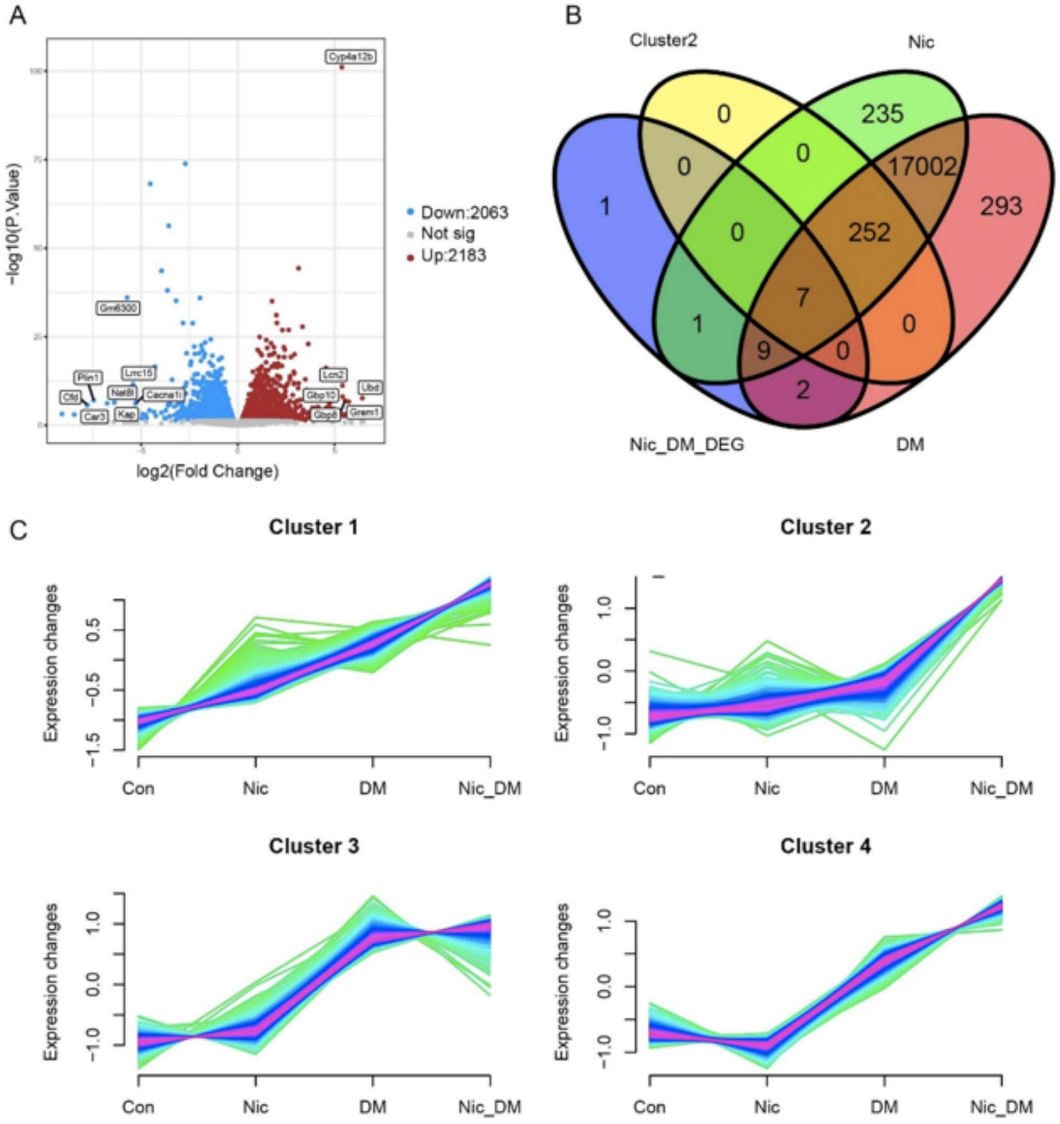
RNA-seq in the kidneys of Con and Nic, DM, Nic + DM mice were analyzed. **A** Differentially expressed genes (DEGs) in Nic + DM group vs. Con group. Red and blue dots represent up- and down-regulated mRNAs, respectively. Upregulated genes that log_2_FC >  = 5 and − lg (p-value) > 5 or the down-regulated genes that log_2_FC <  = − 5 and − lg (p-value) > 5 were listed. **B** Venn analysis of the top 50 genes with the highest fold up-regulation, all genes in cluster 2, and all expressed genes identified in the Nic and DM groups. **C** Fuzzy C-means clustering was performed to identify groups of particular genes responsive to nicotine-exacerbated DN in mice

To uncover the underlying molecular mechanisms for nicotine-exacerbated DN, we performed RNA-seq analysis to compare the transcriptomes among the treatments of control (Con), nicotine (Nic), diabetic mellitus (DM) group, and combination of nicotine and diabetic mellitus (Nic + DM). We randomly selected three mice from each group and extracted total RNAs from their kidneys to construct sequencing libraries. We obtained about 2.95 G of clean data with an average mapping rate of 95.76% per sample to the reference genome (data not shown). A total of 20,110 genes were identified in all samples (Additional file 3: Table S1). To confirm the effects of different treatments on gene expression in mouse kidneys, we analyzed the gene expression between the experimental groups (Nic, DM, and Nic + DM) and the control group. Compared with the control group, 4246 differentially expressed genes (DEGs) were identified in the Nic + DM group, of which 2063 genes were down-regulated, and 2183 were upregulated. (Fig. 7A, Additional file 4: Table S2). To explore the genes strongly reflected in the process of nicotine-promoting DN damage, the parameters were selected as log_2_FC >  = 5 (upregulated) or log_2_FC <  = − 5 (down-regulated) but − log10 (p-value) > 5 at the same time. We found that five upregulated genes (Cyp4a12b, Lcn2, Gbp10, Gbp8, Ubd, Grem1) and eight down-regulated genes (Gm6300, Plin1, Cfd, Car3, Kap, Nat8l, Lrrc15, and Cacna1i) were strongly responsive to the nicotine promoting DN damage (Fig. 7A).

We also analyzed DEGs in the Nic group vs. Con group and the DM group vs. Con group, respectively. Compared with the control group, we identified that 1610 genes were down-regulated and 1632 genes were upregulated in the Nic group (Additional file 2: Fig. S2A, Additional file 5: Table S3), 755 genes were down-regulated, and 936 genes were upregulated in the DM group (Additional file 2: Fig. S2B, Additional file 6: Table S4). We found that Gm6300 and upregulated genes Ubd, Gm14391, and Art2a-ps strongly responded to hyperglycemia (Additional file 2: Fig. S2B). These genes presented as a differential expression in the DM and Con groups. The Gm6300 and Ubd genes were also obviously differentially expressed in the Nic + DM group vs. Con group. These two genes may be specific genes involved in regulating hyperglycemia damaging kidneys. Moreover, we found that some genes related to oxidative stress were differentially expressed. For example, CYBB and Ncf1, which promote ROS production, were upregulated. At the same time, SOD1, which inhibits oxidative stress, was significantly down-regulated in the Nic + DM group vs. the Con group (Additional file 4: Table S2).

### Special gene group responded in nicotine-exacerbated DN mice

We performed a Fuzzy C-means clustering analysis to determine the genes playing important roles in nicotine and hyperglycemia-induced kidney damage. Our results showed that 1082 genes clustered into four unique expression patterns in 4 groups of samples (Fig. 7C, Additional file 7: Table S5). Interestingly, 259 genes were clustered into cluster 2, and the expression patterns of these genes presented an exciting phenomenon (Additional file 7: Table S5). The overall expression trend of these genes is that the expression levels of the first three groups (Con, Nic, and DM) are relatively close. However, there is a sudden up-regulation trend in the fourth group (Nic + DM) (Fig. 7C). The upregulated genes in Nic + DM vs. Con and the fold change (FC) were much larger than those in DM vs. Con; candidate genes were selected for Fuzzy C means clustering analysis. These 259 genes of cluster 2 may be a potential hub genome that specifically increases and responds to nicotine-induced DN kidney damage nicotine to promote DN damage.

Further, we performed the Venn analysis to identify genes involved in nicotine-promoting kidney damage in DN. The selected 50 genes had the highest up-regulation levels, were in cluster 2, and were placed in both the Nic and the DM groups. Venn diagram showed seven common genes (Ubd, Saa1, Grem1, Gbp8, Lcn2, Mat1, Chil3) among the four treatment groups (Fig. 7B). These genes might play critical roles in the process of nicotine and high glucose-induced kidney damage.

### Grem1 may be a vital gene in nicotine exacerbating mouse DN

**Fig. 8:**
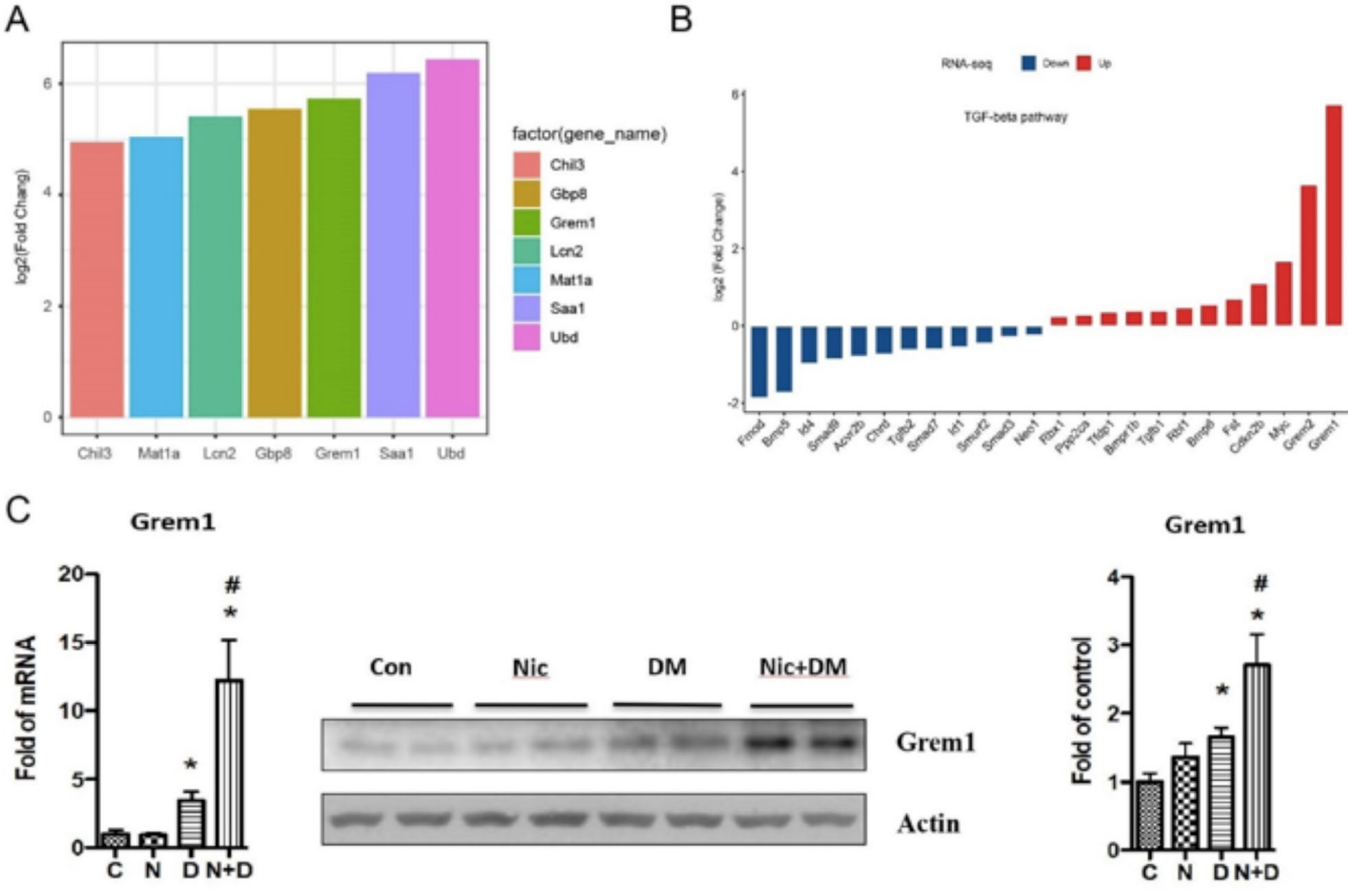
Grem1 responded to the nicotine and hyperglycemia induced in the kidneys of mice. **A** Fold change (FC) of 7 common genes in Nic + DM group vs. Con group. **B** DEGs in the Nic + DM vs. Con group were mapped to the TGF-β pathway. **C** Western blot (middle and right) and real-time PCR (left) analysis of Grem1 protein and mRNA levels in the kidneys of Con and Nic, DM, Nic + DM mice. Grem1 mRNA expression from four independent groups of samples was analyzed through real-time PCR (left); Representative gels were displayed, protein bands scanned (middle), and acquired images were analyzed for data quantification using the NIH image program in the public domain (right). Results (mean ± SD) are representative of three independent samples. *(p < 0.05) compared with the Con group, and # (p < 0.05) compared with the DM group. Note: C, control; N, nicotine; D, diabetes mellitus; N + D, nicotine + diabetes mellitus

To identify genes that play a vital role in nicotine-exacerbated DN in mice, we first screened the fold changes (FC) of the 7 common genes in the Nic + DM group compared to the Con group. Our results showed that Ubd, Saa1, and Grem1 were the top three most upregulated genes (Fig. 8A). We also analyzed the expression trends of 7 common genes in the three comparison groups, including Nic + DM vs. Con, Nic + DM vs. Nic, and Nic + DM vs. DM. Surprisingly, only Grem1 of the top three genes (Ubd, Saa1, and Grem1) was upregulated in the three comparison groups (adopted p value < 0.05, Table 2). Furthermore, our data showed that Grem1 (log_2_FC = 2.854042328) and Grem2 (log_2_FC = 2.508648596) genes were also upregulated in the DM vs. Con group (Additional file 6: Table S4). However, the difference in up-regulation was not significant. However, Grem1 (log_2_FC = 5.731750645) was significantly upregulated than Grem2 (log_2_FC = 3.655715778) in the DM + Nic vs. Con group (Additional file 4: Table S2).

**Table 2:** The fold changes of identified seven differentially expressed genes in three groups

Gene id	Gene name	Nic + DM vs. Con		Nic + DM vs. Nic		Nic + DM vs. DM	
		log2FC	p-value	log2FC	p-value	log2FC	p-value
24108	Ubd	6.4330023	2.14E−08	5.568934739	3.42102E−06	1.405613845	0.260412665
20208	Saa1	6.191799165	0.000046	3.271862017	0.009054783	1.43047504	0.159121216
23892	Grem11	5.731750645	0.000000226	5.001079469	4.89209E−07	2.883274271	0.002156905
76074	Gbp8	5.54548219	0.000000125	3.928375351	6.72766E−05	1.457002581	0.158508517
16819	Lcn2	5.40400181	5.93E−12	5.181678867	1.61987E−11	2.290049156	0.02696154
11720	Mat1a	5.043812868	0.003280271	2.448511208	0.094333698	1.361990523	0.47284171
12655	Chil3	4.947779857	0.0000217	3.158978799	0.000422072	2.538680922	0.012031274

Grem1 is a critical member of the TGF-β signaling pathway (Marquez-Exposito et al. 2020), so we suspect that the TGF-β signaling pathway involves nicotine-promoting kidney damage in DN. To test this hypothesis, we analyzed the DEGs in the Nic + DM vs. Con group. We found that 24 DEGs were mapped to the TGF-β pathway, including 12 down-regulated and 12 upregulated genes (Fig. 8B). It is worth noting that Grem1 was still the gene with the most significant fold change among the 24 DEGs (Fig. 8B). Notably, the Grem2 gene was the second most upregulated gene mapped to the TGF-β pathway. These results suggest that the 24 DEGs represented by Grem1 in the TGF-β signaling pathway were critical in regulating nicotine to promote kidney damage in DN. Accordingly, the TGF-β pathway is likely one of the core pathways involved in nicotine-exacerbated DN.

To confirm the expression of Grem1 in nicotine-exacerbated DN, real-time PCR, and Western blot were performed to examine its expression in mouse kidneys. Our results showed that nicotine had no significant effect on Grem1 expression in mice with blood glucose levels less than 200 dg/mL; however, it significantly increased Grem1 expression in the kidneys of diabetic mice (Fig. 8C).

**Fig. 9:**
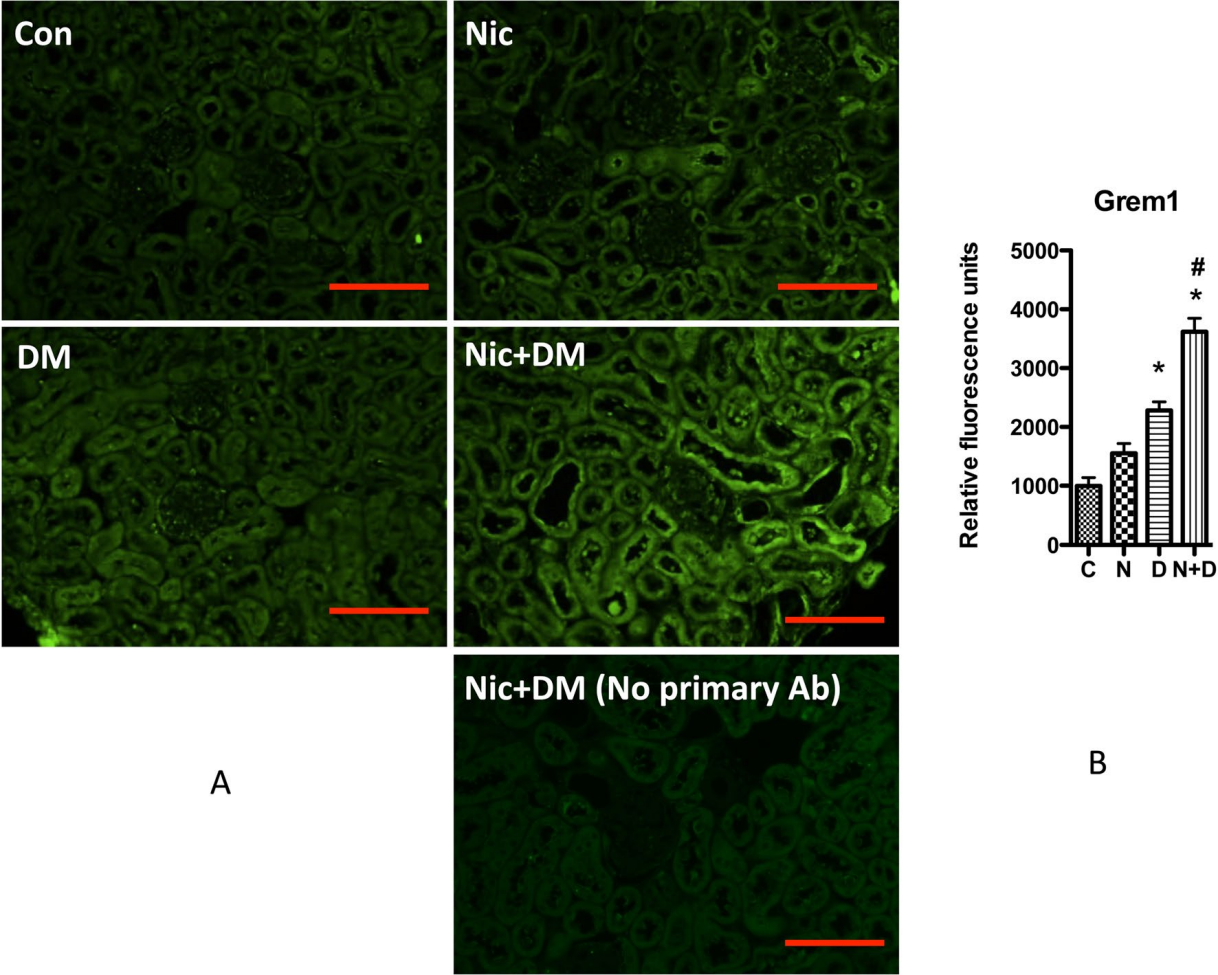
Grem1 expression in the kidney cortex of mice. **A** Immunofluorescence staining was performed to detect the Grem1 expression in the kidneys of Con, Nic, DM, and Nic + DM mice. A negative control without a primary antibody was used to show the image background. The scale bar is 50 μm. **B** The average fluorescence intensity was measured per region of interest (ROI) for Grem1, and the results (mean ± SD) represent 10 randomly and blindly selected regions. * (p < 0.05) compared with the Con group, and # (p < 0.05) compared with the DM group. Note: C, control; N, nicotine; D, diabetes mellitus; N + D, nicotine + diabetes mellitus

**Fig. 10:**
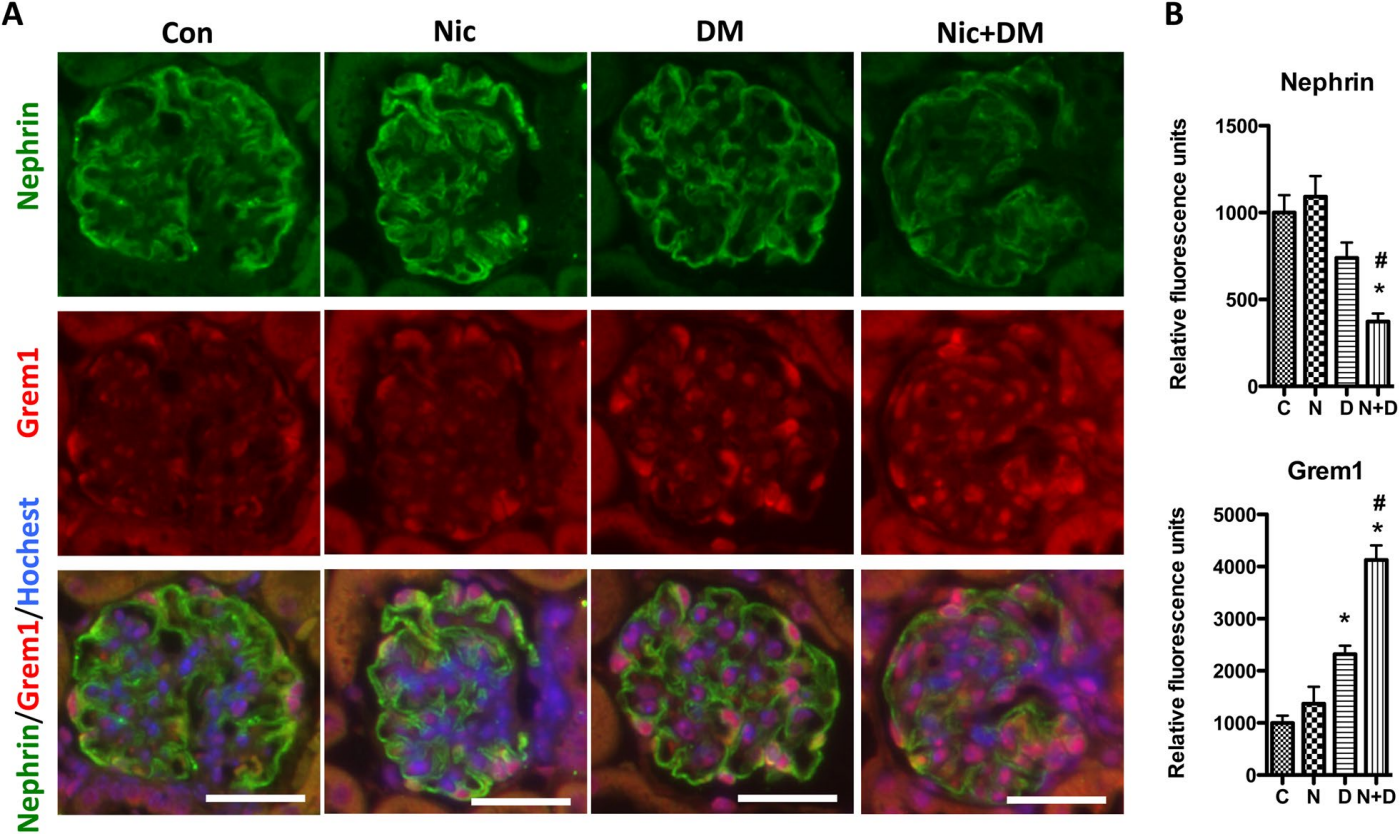
Grem1 expression in the glomerular of mouse kidneys. Immunofluorescence staining was performed to determine the co-localization of Grem1 and Nephrin in mouse glomeruli. **A** Co-localizations of Grem1 and Nephrin in representative figures. The scale bar is 20 μm. **B** The average fluorescence intensity was measured per region of interest (ROI) for Grem1 or Nephrin, and the results (mean ± SD) represent 10 randomly and blindly selected regions. *(p < 0.05) compared with the Con group, and # (p < 0.05) compared with the DM group. Note: C, control; N, nicotine; D, diabetes mellitus; N + D, nicotine + diabetes mellitus

In addition, we further confirmed the expression of Grem1 in mouse kidneys through immunofluorescent staining. As displayed in Fig. 9, nicotine increased Grem1 expression in the glomerular and interstitium under hyperglycemia. Considering the importance of podocytes for kidney function, we also examined Grem1 expression in podocytes by immunofluorescence staining. The results showed that some Grem1-expressing cells also expressed nephrin, a molecular marker for podocytes, indicating that nicotine could increase Grem1 expression in podocytes (Fig. 10).

### The phosphorylation of the Smad signaling pathway, downstream of the TGF-β pathway, responds to the nicotine and exacerbates hyperglycemia-induced Grem1 increasing

**Fig. 11:**
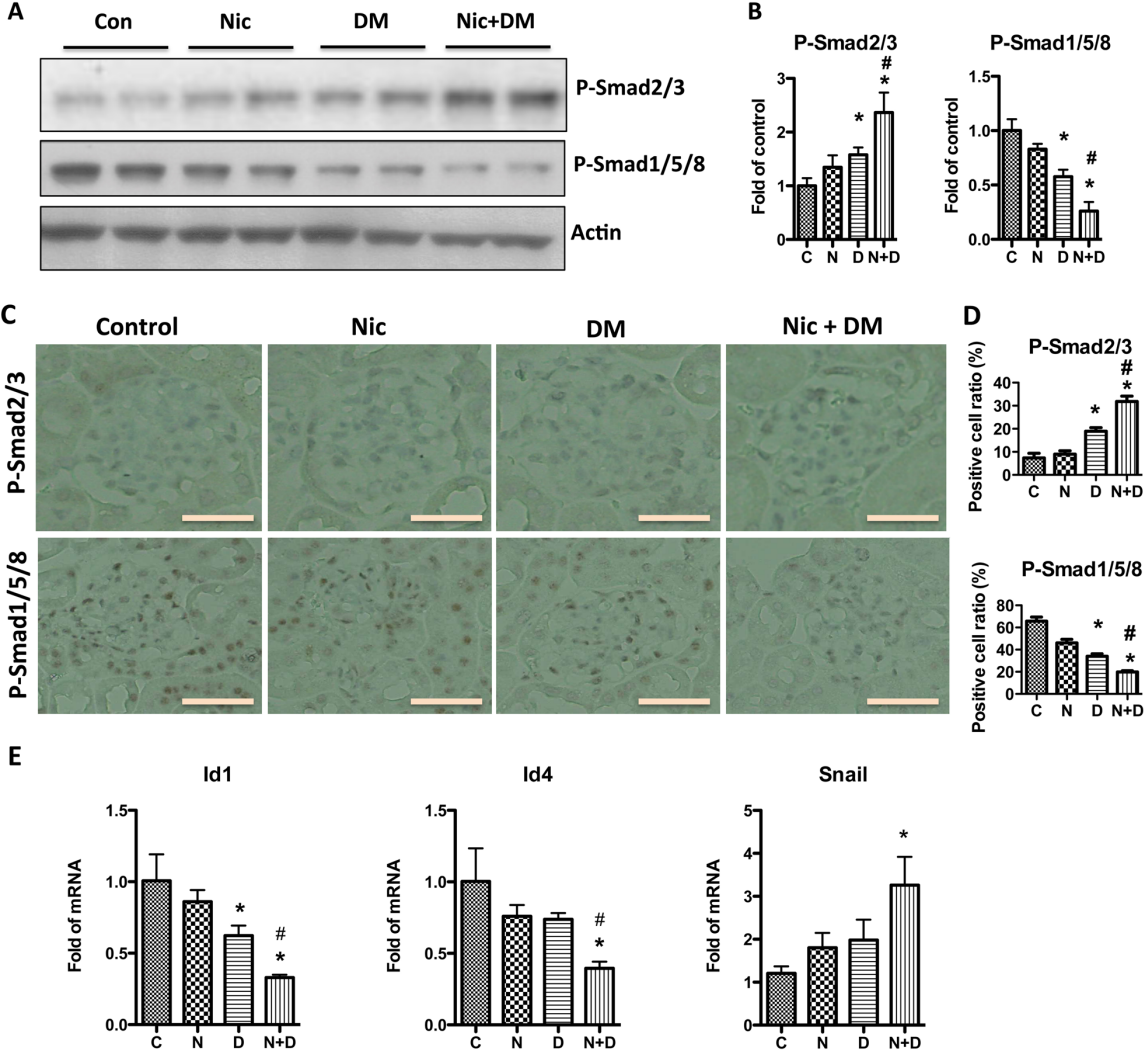
Nicotine increases the phosphorylation status of Smads, Ids, and Snail in mouse kidneys. **A** Western blot was performed to investigate the expression of p-Smad2/3 and p-Smad1/5/8 in the kidneys of Con, Nic, DM, and Nic + DM mice, and the representative gels are displayed. **B** The acquired images were used for data quantification, and the expression of p-Smad2/3 and p-Smad1/5/8 was normalized to β-actin. Results (mean ± SD) are representative of three independent samples. **C**, **D** Immunohistochemical staining of p-Smad2/3 and p-Smad1/5/8 in kidneys of Con and Nic, DM, Nic + DM mice. The scale bar is 20 μm. The average fluorescence intensity was measured per region of interest (ROI) for p-Smad2/3 and p-Smad1/5/8, and the results (mean ± SD) represent 10 randomly and blindly selected regions. **E** RNAs were extracted, and real-time PCR was performed to detect Id1, Id4, and Snail mRNA expression in the kidneys of Con and Nic, DM, and Nic + DM mice. Results (mean ± SD) are representative of three independent samples. * (p < 0.05) compared with Con group and # (p < 0.05) compared with DM group. Note: C, control; N, nicotine; D, diabetes mellitus; N + D, nicotine + diabetes mellitus

Grem1 plays its function through 3 signaling pathways: (1) activation of Smad2/3; (2) suppression of Smad1/5/8; (3) activation of VEGFR2. We examined the effects of nicotine and hyperglycemia on these downstream signaling pathways. Western blotting and IHC staining showed that the combination of nicotine and hyperglycemia significantly increased the activation of Smad2/3 and decreased the activation of Smad1/5/8 (Fig. 11A–D) compared with nicotine or hyperglycemia alone.

To further confirm the activity changes of Smads, we examined the mRNA of Id1 and Id4, the targeting genes for Smad1/5/8, and Snail, the targeting gene for Smad2/3. The results showed that the combination of nicotine and hyperglycemia significantly decreased the mRNA levels of Id1 and Id4 but increased Snail (Fig. 11E). At the same time, our RNA-seq data also showed that the Id1 and Id4 were down-regulated, and Snail was upregulated in Nic + DM vs. Con group (Additional file 4: Table S2). These data suggest that nicotine promotes the activation of Smad2/3 but suppresses Smad1/5/8 in hyperglycemia.

### Knocking down Gram1 partially attenuated nicotine and high glucose-induced podocyte injury

**Fig. 12:**
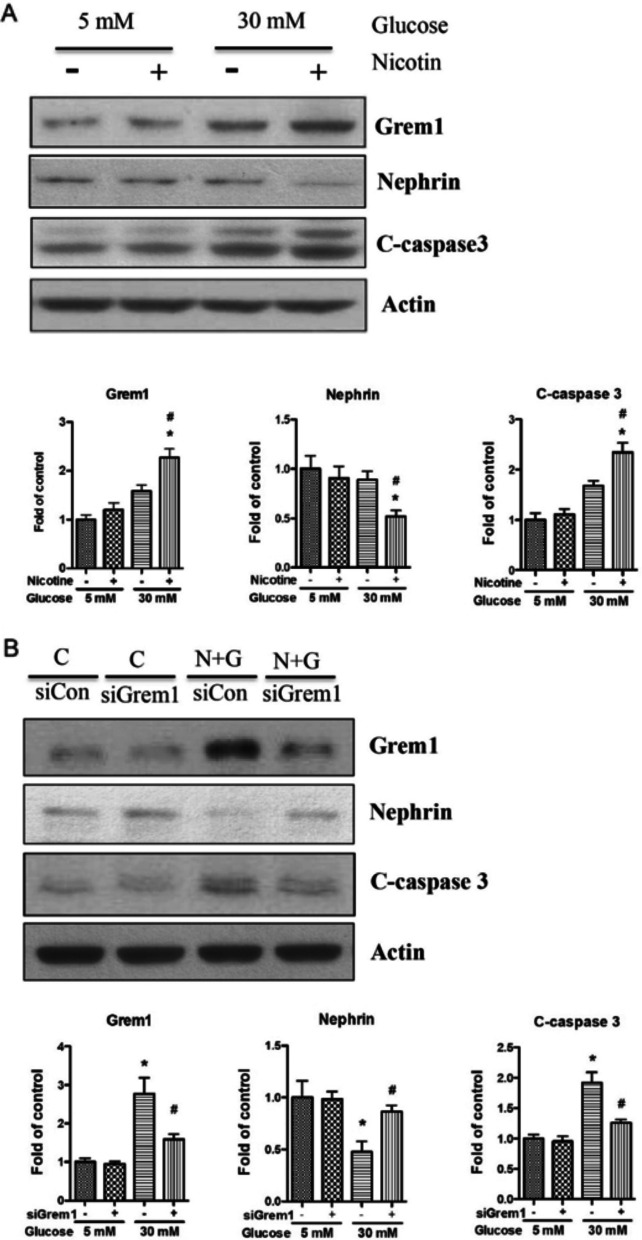
Knocking down Gram1 partially attenuated nicotine and high glucose-induced podocyte injury. **A** Western blot was performed to investigate the expression of Grem1, nephrin, and cleaved caspase 3 in podocytes co-treated with nicotine and high glucose. **B** Knocking down Grem1, a Western blot was performed to investigate the expression of Grem1, nephrin, and cleaved caspase 3 in podocytes co-treated with nicotine and high glucose. The acquired images were used for data quantification, and the expression of Grem1 was normalized to β-actin. Results (mean ± SD) are representative of three independent samples. *(P < 0.05) in comparison with control (5 mM glucose, 0 μM nicotine) and #(P < 0.05) in comparison with nicotine treatment (5 mM glucose, 10 μM nicotine). Note: C-caspase3, cleaved caspase3

To examine the role of Grem1 in nicotine-exacerbated DN, we treated human podocytes with high glucose and nicotine. The results showed that high glucose and nicotine co-treatment significantly increased Grem1 expression (Fig. 12A). Cleaved caspase 3, a biomarker of cell apoptosis, was also raised with nicotine and high glucose; however, the expression of nephrin was significantly decreased (Fig. 12A).

To establish the causation between Grem1 expression and podocyte injury, we knocked down Grem1 expression with its specific siRNA. The results showed that knocking down Grem1 expression significantly reduced cleaved caspase 3 but partially restored nephrin expression (Fig. 12B). These results demonstrated that Grem1 was vital in nicotine-exacerbated podocyte injury in high glucose milieu.

## Discussion

Cigarette smoking is among the top ten contributors to global morbidity and mortality (Lande et al. 2010). Increasing evidence shows that cigarette smoking is an independent risk factor for developing and progressing chronic kidney disease, including DN (Jain and Jaimes 2013; Hall et al. 2016; Bello et al. 2017; Xia et al. 2017; Zhu et al. 2017). Therefore, understanding the relationship between smoking and DN is significant for preventing and treating DN. In this study, we focused on the role of nicotine, the primary active component of cigarette smoke, in the progress of DN. According to a clinical report, the concentrations of cotinine, the primary metabolite of nicotine in the plasma of smokers, were 21–4420 ng/mL with an average of 379.4 ng/mL (Massadeh et al. 2009), close to the maximal concentrations (204 to 364 ng/mL) of cotinine in the blood of three inbred strains of mice after IP injection of nicotine at 1 mg/kg (Petersen et al. 1984). Another study demonstrated that the plasma level of cotinine was significantly higher in female (572.7 ± 66.9 ng/mL) than in male (472.8 ± 44.0 ng/mL) mice after receiving nicotine injection at 1 mg/kg (Nguyen et al. 2020). The present study specifically focused on female mice to attain a higher cotinine concentration in the plasma. These mice were administered nicotine at 1 mg/kg via intraperitoneal (IP) injection. Remarkably, we observed that the mean plasma cotinine concentration reached 349.5 ng/ml ± 81.0 ng/ml, which closely approximates the average level found in smokers. Our results showed that nicotine alone did not cause apparent kidney damage. Nonetheless, it significantly boosted hyperglycemia-induced albuminuria, BUN, plasma creatinine, kidney tissue mRNA expression of Kim and NGAL, glycogen and collagen accumulation, and apoptosis of kidney cells, suggesting that nicotine can potentially exacerbate DN. Through RNA-seq analysis, we identified the role of Grem1 and its related TGF-β signaling pathway in the development and progression of DN. Additionally, in vitro data showed that suppressing Grem1 expression could partially attenuate nicotine-exacerbated podocyte injury. The present study demonstrated that nicotine exacerbates DN by upregulating Grem1 expression. This is the first report to reveal one of the molecular mechanisms involved in smoke-mediated exacerbation of DN.

To systematically understand the molecular mechanisms for cigarette smoke-enhanced DN, we treated mice with nicotine (Nic), hyperglycemia (DM), and the combination of nicotine and hyperglycemia (Nic + DM). Then we performed RNA-seq to compare the kidney transcriptome changes of these mice. Compared with the control group (Con), we identified 3242, 1691, and 4246 DEGs in the Nic, DM, and Nic + DM groups, suggesting that nicotine is involved in regulating this process by affecting a wide range of gene expression in the process of aggravating the kidney damage in the diabetic milieu. Among the DEGs induced by nicotine and hyperglycemia, we identified Grem1 as the most notable in all the three comparison groups (Nic + DM vs. Con, Nic + DM vs. Nic, and Nic + DM vs. DM) (Table 2). Grem1, a member of the DAN family, represents a collection of BMP antagonists that are highly expressed during development and have important roles in limb bud formation and digitation, kidney formation and morphogenesis, and left–right axis specification (Zúñiga et al. 1999; Khokha et al. 2003; Marques et al. 2004; Michos et al. 2004, 2007). Previous studies have shown that overexpression of Grem1 could lead to aggravation of kidney damage. For example, in an experimental mouse with folic acid-induced nephropathy, specific overexpression of Grem1 in tubules increased renal impairment, which was mainly mediated by up-regulation of local proinflammatory and pro-fibrotic factors (Droguett et al. 2014). Similar results were found in studies of STZ-induced DN in a mouse model (Marchant et al. 2015). In addition, in human DN, the expression level of Grem1 is positively correlated with the renal tubulointerstitial fibrosis score, suggesting that Grem1 has a deleterious effect on human kidney disease (Dolan et al. 2005). Our study also showed that nicotine increased Grem1 expression in renal interstitium, glomeruli, and podocytes. It is well known that Grem1 is a gene involved in the TGF-β signaling pathway. By comparing the differentially expressed genes with genes in the TGF-β signaling pathway from the KEGG database, we found that 24 DEGs were mapped to the TGF-β pathway (Fig. 8B), of which Grem1 and Grem2 were the top 2 upregulated genes. Although previous studies by our group demonstrated that Grem2 increasing induced by high glucose could mediate podocyte apoptosis (Wen et al. 2019), and indeed we also found that the Grem1 and Grem2 were both upregulated in the DM vs. Con group; however, the present findings showed that Grem1 was the most responsive to nicotine-exacerbated kidney damage in DN compared to Germ2. The results suggest that Grem1 may be a significant gene in the TGF-β pathway involved in nicotine-exacerbated kidney damage in DN. Furthermore, we confirmed the role of TGF-β signaling in nicotine-mediated kidney damage. We found that the combination of nicotine and hyperglycemia increased the activation of Smad2/3 and the expression of its target genes Id1 and Id4 but decreased the activation of Smad1/5/8 and the expression of its target gene Snail (Fig. 11). Similarly, we confirmed in our RNA-seq data that Id1 and Id4 were down-regulated. However, Snail was upregulated in Nic + DM vs. Con group (Additional file 4: Table S2). The present study demonstrated that nicotine-mediated diabetic kidney injury involves the TGF-β signaling pathway in general and Grem1 in particular.

Recently, in a mouse model similar to human crescentic nephritis induced by nephrotoxic serum (NTS), treatment with bromodomain and extra-terminal domain (BET) inhibitor (iBET) in NTS-injected mice inhibited renal Germ1 overexpression and diminished glomerular damage, thereby restoring podocytes, suggesting that inhibition of Germ1 has significant potential value in nephritis (Tejedor-Santamaria et al. 2022). Notably, the present study showed that the knockdown of Grem1 significantly restored nephrin expression under combined treatment with nicotine and high glucose (Fig. 12B). This implies that Grem1 inhibition might ameliorate podocyte damage by nicotine and high glucose. In summary, the present study confirms that Grem1 acts as a critical gene, and participation of the TGF-β signaling pathway is significant in nicotine-exacerbated kidney damage in the diabetic milieu. It is possible that interfering with the expression of Grem1 will be an effective way to treat long-term diabetic smokers, and Grem1 may also become a potential target for developing related therapeutic drugs. It will be interesting to generate knockout mice to remove/reduce Grem1 expression and expose them to nicotine and hyperglycemia alone or in combination. By comparing the kidney injuries induced by different milieus, we can further confirm the role of Grem1 in the progress of nicotine-exacerbated DN.

Clinical studies have shown a strong association between smoking and chronic kidney disease (CKD) progression in patients with diabetes, hypertension, polycystic kidney disease, and kidney transplantation. The renal effects of nicotine are closely related to the increased production of reactive oxygen species (ROS) and the activation of pro-fibrotic pathways, which are involved in many signaling pathways such as TGF-β, NADHP, Akt/PKB and NOX4-mediated ROS production (Jain and Jaimes 2013). Our studies have demonstrated that nicotine and high glucose-mediated oxidative stress contributed to kidney cell injury (Lan et al. 2016; Jiang et al. 2019; Wen et al. 2019). NADPH oxidases (Nox) is a membrane-bound enzyme complex that generates superoxide, a substrate for subsequent reactions that cause ROS. From the RNA-seq results of this study, we found that nicotine administration significantly increased the gene expression of some NADPH-oxidase subunits, such as CYBB and Ncf1. In contrast, nicotine further increased the hyperglycemia-induced expression of Ncf1 (Additional file 4: Table S2). Moreover, it has been reported that the deficiency of superoxide dismutase (SOD1) in mice decreases antioxidant enzyme activities and produces severe oxidative stress (Hwang et al. 2020). The present study also showed that the SOD1 was significantly down-regulated by nicotine in DN (Additional file 4: Table S2). These results strongly support the previous findings that increased oxidative stress caused by nicotine and/or high glucose contributes to the progress of DN. It will be interesting to examine the synergistic effect of increased oxidative stress caused by nicotine and high glucose and to investigate whether Grem1 regulates the expression of related genes.

It has been reported that tobacco smoking influences Grem1 expression. For example, Al-Najeem et al. said that there was a significant increase (p < 0.05) in Grem1 serum levels in smokers compared with nonsmokers (Al-Najeem and Al-Dujaili 2017). However, in this study, we didn't see nicotine alone significantly increase the expression of Grem1. One possible reason for this difference is that we examined the Grem1 expression only in the kidney but not in other organs. It will be interesting to determine the Grem1 expression in the serum of these mice treated with nicotine only.

In addition to Grem1, other factors, such as elevated blood pressure (BP), may contribute to nicotine-exacerbated DN. It is well known that high BP (hypertension) is one of the main contributors to chronic kidney injury (Mennuni et al. 2014; Tian and Liang 2021). As nicotine administration can increase BP in mouse models (Backer and Schlager 1984; Oakes et al. 2020; Wang et al. 2021), the increased BP might contribute to barotrauma and kidney injury. In a rat model of warm ischemia–reperfusion, the administration of nicotine (200 μg/ mL in drinking water) resulted in worse morphological changes and renal function; however, administrating nicotine to sham-operated rats also increased oxidative stress without significant morphological changes (Jain and Jaimes 2013). Consistent with this report, nicotine administration alone didn't cause apparent kidney injury in our study either. These results suggest nicotine-increased BP was not an independent factor causing kidney injury. However, after long-term hyperglycemia, the kidney becomes more vulnerable and sensitive to BP changes. Whether nicotine-elevated BP and STZ-induced hyperglycemia formed a synergistic effect to cause kidney injury requires further investigation. If nicotine treatment early after STZ administration does not induce a synergistic effect on kidney injury, it would suggest the kidney is primed for barotrauma.

This study used STZ injection to generate the hyperglycemia mouse model. STZ injection could partially damage the islets, thus triggering an inflammatory response, leading to further loss of β cell function, insulin deficiency, and hyperglycemia (Noshahr et al. 2020). Although STZ injection is one of the most commonly used methods to generate diabetes models, this method still has some limitations, such as unstable induction of hyperglycemia, the possibility of using endogenous mouse islets to restore normoglycemia and STZ toxicity (Gvazava et al. 2018; Heather et al. 2022). These limitations make it unable to simulate the changes observed in DN in humans perfectly. To better mimic diabetes mellitus in humans, other models, such as genetically induced insulin-dependent diabetes models and type 2 diabetes obese models, should also be considered in further studies.

## Conclusion

Nicotine exacerbates DN through the upregulation of Grem1 expression in kidney cells. The present study reveals a new molecular mechanism for nicotine-enhanced kidney injury. It highlights novel potential therapeutic targets for smoking-related DN.

## Supplementary Information

**Additional file 1: Figure S1**. Schematic diagram of the mouse administration procedure.

**Additional file 2: Figure S2**. RNA-seq identified the differentially expressed genesin kidneys.Differentially expressed genesin the Nic and Con groups.DEGs in DM group vs. Con group. Red and blue dots represent up- and down-regulated mRNAs, respectively. Upregulated genes that log2FC >  = 5 and − log10 > 5 or the down-regulated genes that log2FC <  = − 5 and log2FC >  = 5were listed.

**Additional file 3: Table S1.** Gene expression level matrix in 12 samples identified by RNA-seq sequencing analysis.

**Additional file 4: Table S2.** The identified up and down-regulated genes in Nic+DM group vs Con group mice by RNA-seq.

**Additional file 5: Table S3.** The identified up and down-regulated genes in Nic group vs Con group mice by RNA-seq.

**Additional file 6: Table S4.** The identified up and down-regulated genes in DM group vs Con group mice by RNA-seq.

**Additional file 7: Table S5.** The identified genes in cluster 1–4.

## Data Availability

The datasets used and/or analyzed during the current study are available from the corresponding author upon reasonable request.
