# Supplementary figures and images for: Nicotine exacerbates diabetic nephropathy through upregulation of Grem1 expression
Source: Mol Med. 2023 Jul 6;29:92. doi: 10.1186/s10020-023-00692-9 (PMC10327355; doi:10.1186/s10020-023-00692-9)

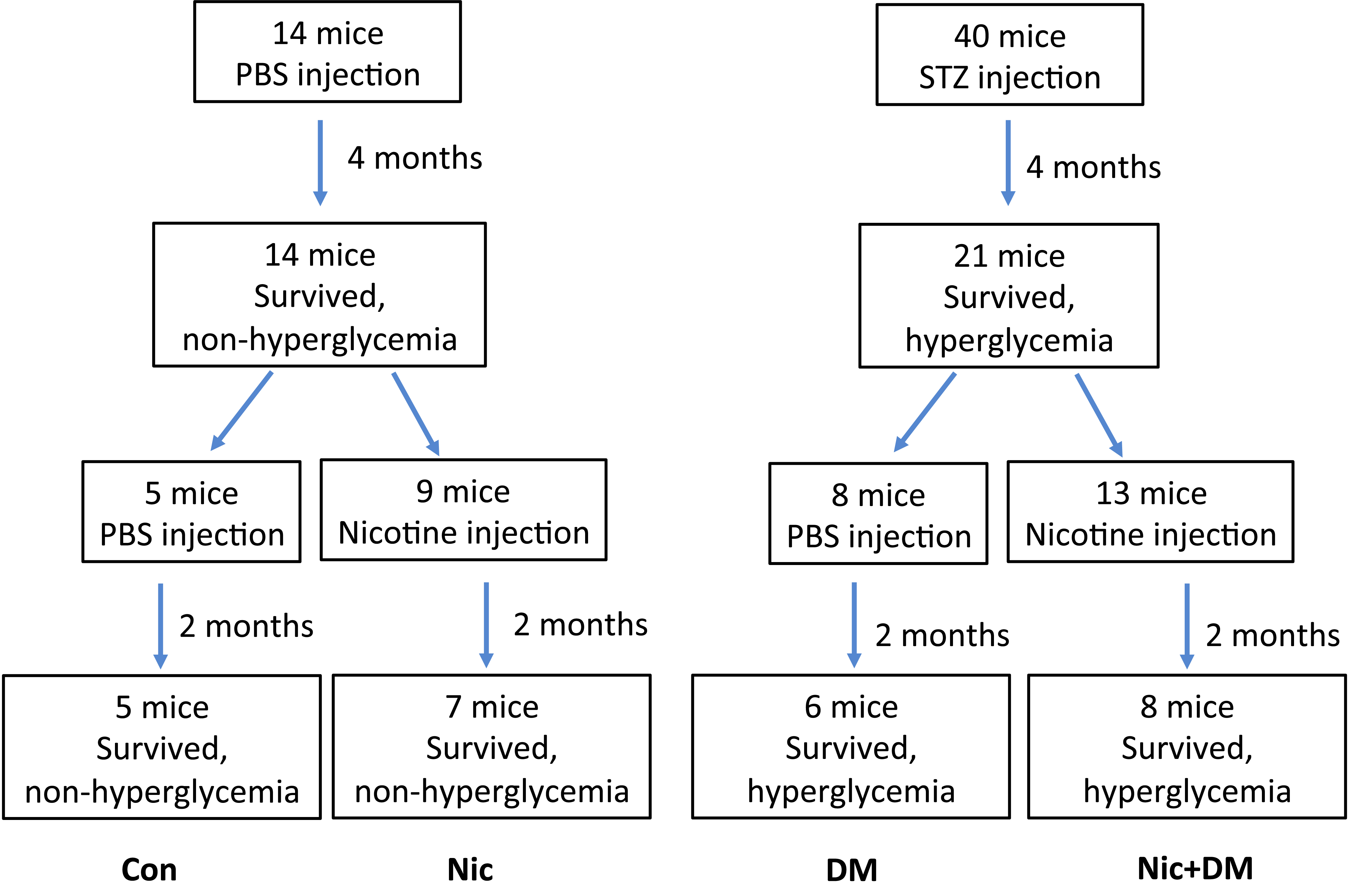

Supplement: Supplementary file 1 — Additional file 1: Figure S1. Schematic diagram of the mouse administration procedure. [file 10020_2023_692_MOESM1_ESM.jpg]

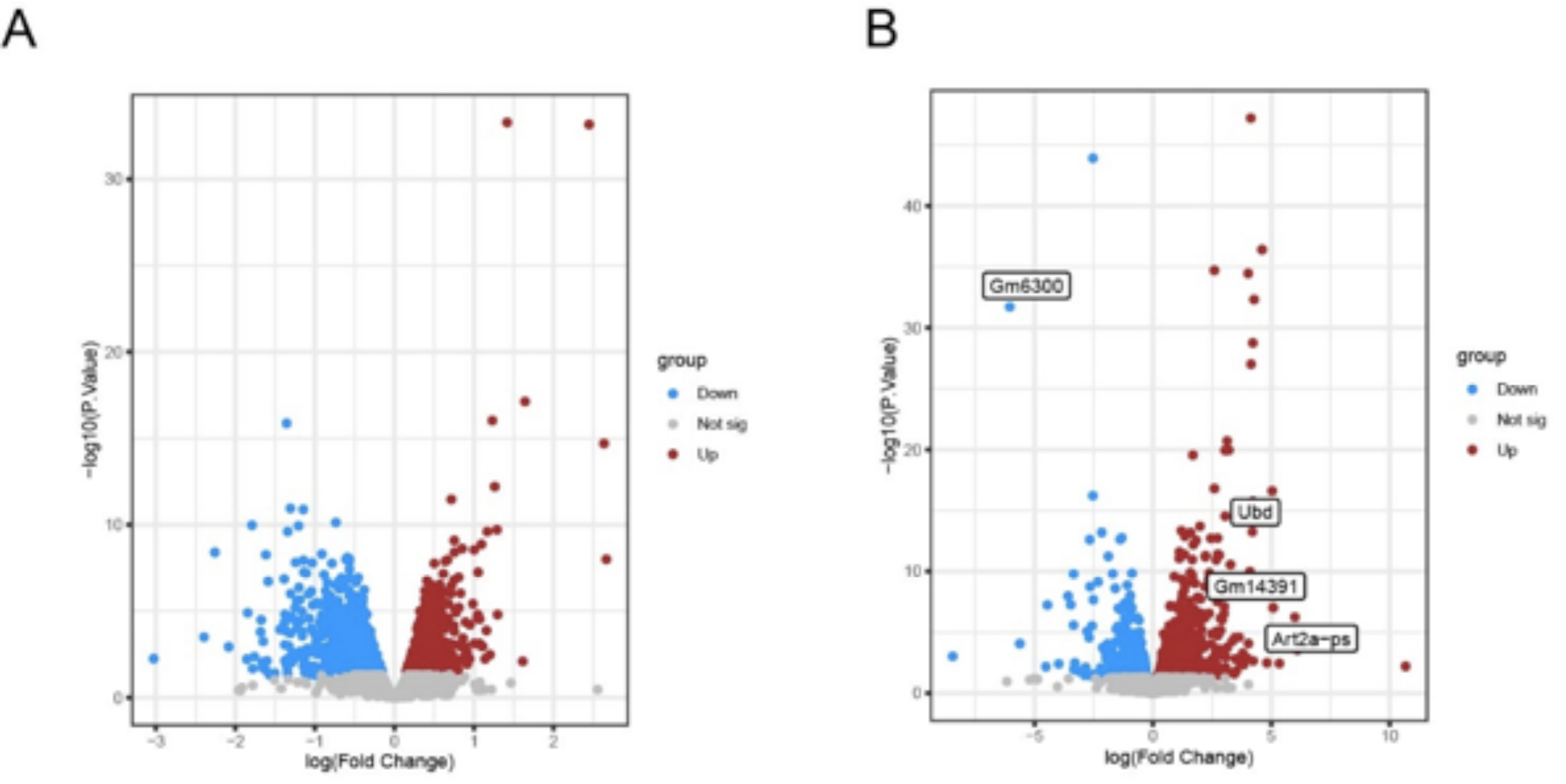

Supplement: Supplementary file 2 — Additional file 2: Figure S2. RNA-seq identified the differentially expressed genesin kidneys.Differentially expressed genesin the Nic and Con groups.DEGs in DM group vs. Con group. Red and blue dots represent up- and down-regulated mRNAs, respectively. Upregulated genes that log2FC > = 5 and − log10 > 5 or the down-regulated genes that log2FC < = − 5 and log2FC > = 5were listed. [file 10020_2023_692_MOESM2_ESM.jpg]
